# Review: corneal endothelial cell derivation methods from ES/iPS cells

**DOI:** 10.1186/s41232-019-0108-y

**Published:** 2019-10-03

**Authors:** Shin Hatou, Shigeto Shimmura

**Affiliations:** Department of Ophthalmology, Keio University School of Medicine, 35 Shinanomchi, Shinjuku-ku, Tokyo, Japan

**Keywords:** Embryonic stem cells, Induced pluripotent stem cells, Neural crest cells, Epithelial-mesenchymal transition, Transforming growth factor beta, Bone morphogenetic protein, Wnt

## Abstract

Globally, approximately 12.7 million people are awaiting a transplantation, while only 185,000 cases of corneal transplantation are performed in a year. Corneal endothelial dysfunction (bullous keratopathy) due to Fuchs’ corneal endothelial dystrophy, or insults associated with intraocular surgeries, shared half of all indications for corneal transplantation. Regenerative therapy for corneal endothelium independent of eye bank eyes has great importance to solve the large supply-demand mismatching in corneal transplantation and reduce the number of worldwide corneal blindness. If corneal endothelial cells could be derived from ES or iPS cells, these stem cells would be the ideal cell source for cell therapy treatment of bullous keratopathy. Four representative corneal endothelial cell derivation methods were reviewed. Components in earlier methods included lens epithelial cell-conditioned medium or fetal bovine serum, but the methods have been improved and materials have been chemically more defined over the years. Conditioned medium or serum is replaced to recombinant proteins and small molecule compounds. These improvements enabled to open the corneal endothelial developmental mechanisms, in which epithelial-mesenchymal and mesenchymal-endothelial transition by TGF beta, BMP, and Wnt signaling have important roles. The protocols are gradually approaching clinical application; however, proof of efficacy and safety of the cells by adequate animal models are the challenges for the future.

## Background

From the data of the Global Survey of Corneal Transplantation and Eye Banking collected between August 2012 and August 2013, approximately 12.7 million people were awaiting transplantation in 134 countries, covering 91% of the world’s population [1]. On the other hand, only 185,000 corneal transplantation were performed in 116 countries [1]. Bullous keratopathy, i.e., corneal endothelial dysfunction due to Fuchs’ corneal endothelial dystrophy or insults associated with intraocular surgeries, shared half of all indications for corneal transplantation [1]. Regenerative therapy for corneal endothelium independent of eye bank eyes may help solve the large supply-demand mismatching in corneal transplantation and reduce the number of worldwide corneal blindness.

The corneal endothelium consists of a single layer of hexagonal cells with a basement membrane (Descemet’s membrane) covering the posterior surface of the cornea in a well-arranged mosaic pattern [2, 3]. Corneal hydration is determined primarily by the balance between the movement of aqueous humor across the corneal endothelium into the stroma and the subsequent pumping of the fluid out from the stroma [2, 3]. The accumulation of fluid in the stroma due to disturbance of this balance may lead to bullous keratopathy, which is characterized by an edematous cornea with a reduced transparency. Tight junction between endothelial cells regulates the movement of aqueous humor across the corneal endothelium into the stroma (barrier function), and the Na^+^- and K^+^-dependent ATPase (Na, K-ATPase) expressed in the basolateral membrane of corneal endothelial cells is primarily responsible for the pump function of the corneal endothelium [2]. Given that human corneal endothelial cells (HCEC) have a limited proliferative capacity, Fuchs’ corneal endothelial dystrophy and insults associated with intraocular surgeries result in corneal endothelial cell loss and permanent damage. Allogenic penetrating keratoplasty (PKP) has been performed for a century, and its low rejection rate is due to anterior chamber-associated immune deviation. Several new corneal endothelial keratoplasty techniques, such as Descemet’s membrane stripping automated endothelial keratoplasty (DSAEK) or Descemet’s membrane endothelial keratoplasty (DMEK), have been clinically performed. Although these techniques are less invasive than PKP, there still remain some problems, such as acute glaucoma attack due to air bubbles in anterior chamber, or host-graft adhesion failure. Long-term endothelial cell loss of the graft requires re-graft operation with another eye bank eye.

Recently, techniques for in vitro culture of HCEC have improved, and a cell injection therapy into the anterior chamber for bullous keratopathy using cultured corneal endothelial cells and Rho-associated kinase (ROCK) inhibitor has been reported [4]. This was the first proof of concept to treat bullous keratopathy by cell suspension therapy. However, the quality of cultured endothelial cells is largely dependent on donor age, so other cell sources which are totally independent from donor cornea is more desirable. On the other hand, several methods to make corneal endothelial cells from embryonal stem cells (ES cells) or induced pluripotent stem cells (iPS cells) have been reported recently. If possible, ES or iPS cells would be the ideal cell source for cell therapy treatment of bullous keratopathy. In this report, represented methods from these articles are reviewed, and future challenges are discussed.

### Development of corneal endothelium

**Fig. 1:**
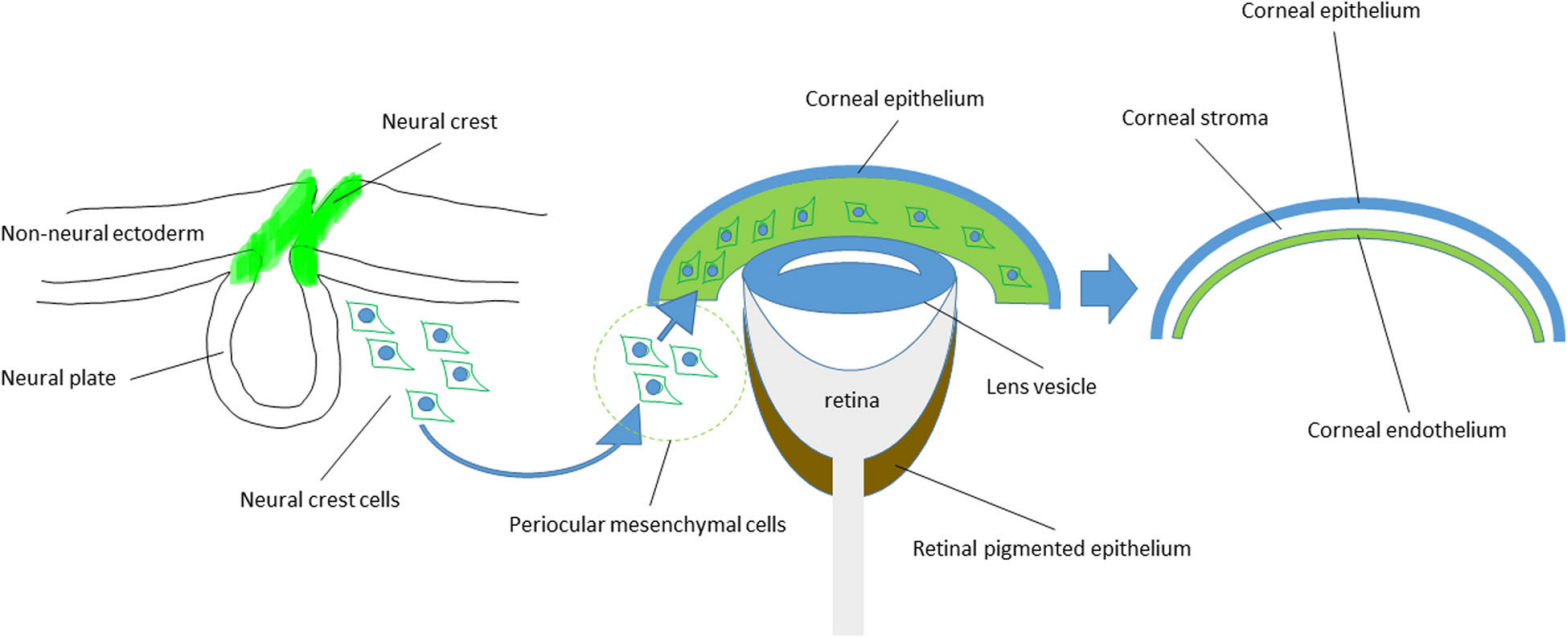
Schema of corneal endothelium development. Neural crest cells begin to migrate from neural plate border with the endothelial-mesenchymal transition and reach periocular lesion. Next, neural crest cells (or periocular mesenchymal cells) migrate beneath corneal epithelium and mature to be corneal endothelium. This process is thought as the mesenchymal-endothelial transition

The anterior segment of the eye is organized from diverse embryological origins, and its mechanism is complicated (Fig. 1). Corneal endothelium and stroma are derived from periocular mesenchyme (POM), or in other words, periocular neural crest cells (NCCs), while corneal epithelium and lens are derived from surface epidermal ectoderm [5]. The cornea is formed at 5–6 weeks of human gestation, when surface ectoderm and lens are completely separated [5]. Mesenchyme cells from neural crest migrate into the space between them and forms several layers [5]. Surface ectoderm becomes corneal epithelium, and the most inner layer mesenchyme becomes corneal endothelium [5]. Next, mesenchyme between corneal epithelium and endothelium becomes the corneal stroma [5]. The endothelial cells become flattened and tightly connected to each other by tight junctions and produce basement membrane which separate endothelium from stroma, called Descemet’s membrane [5]. Finally, corneal endothelial cells form typical hexagonal monolayer, and they stay arrested in the G1-phase of mitosis [5, 6].

The mechanisms of neural crest cell migration and corneal endothelial cell maturation are not fully understood. Neural crest cell migration from neural plate border begins with epithelial-mesenchymal transition (EM-T). They migrate to periocular lesion and subsequently reach the area beneath the corneal epithelium. Transforming growth factor beta (TGF beta) signaling or Wnt signaling may contribute in this process [7, 8]. However, corneal endothelial cells forms typical endothelial morphology with cobble stone appearance, so there may be reversal steps of mesenchymal-endothelial transition (ME-T) in corneal endothelium maturing process. Some of the transcription factors involved in these events are reported: Foxc1, Foxc2, Lmx1b, Pax6, Pitx2, RAR*β*, RAR*γ*, RXR*α*, Six3, and Smad2 [5, 8–12]. However, it has not been clarified which of them works in migration process or which works during the maturing process. Among them, Foxc1, Pitx2, and Pax6 are responsive genes for anterior segment dysgenesis, Peters’ anomaly and Axenfeld-Rieger syndrome [12]. These facts may suggest these transcriptional factors may play a role in migration process.

### Strategies for producing corneal endothelial cells from stem cells

To date, most approaches for producing corneal endothelial cells from ES or iPS cells in vitro were by mimicking developmental process; in the first step, neural crest cells were derived from iPS/ES cells. Next, corneal endothelial cells were derived from neural crest cells. However, the materials and methods vary much with researchers and developers. Four representative methods are reviewed as follows.

#### Co-culture with corneal stroma cells and lens epithelial cell-conditioned medium

Since corneal endothelium is located between corneal stroma and lens and it is organized after lens development, corneal endothelial cells may be produced under the influence of lens epithelial cells and corneal stroma cells. Zhang et al. derived corneal endothelial-like cell from human ES cells by co-culture with human corneal stroma cells and lens epithelial cell-conditioned medium (LECCM) [13]. Table 1 shows the summary of their methods. First, embryoid bodies (EB) were formed in low attachment culture dishes. Next, EB were plated on fibronectin, laminin, and heparin sulfate-coated glass coverslips, and co-cultured with corneal stroma cells which were obtained from Chinese eye bank. Co-culture was started with basal medium containing epidermal growth factor (EGF), basic fibroblast growth factor (bFGF), and fetal bovine serum, and subsequently, the medium was changed to LECCM. LECCM were collected from SV-40-transformed human lens epithelial cell culture medium. After 5 days of co-culture, CD73/FoxC1 co-expressing POM cells emigrated from EB. N-cadherin/vimentin dual positive corneal endothelial-like cells were obtained after LECCM culture. Na,K-ATPase alpha-1 and beta-1 subunits were upregulated in their corneal endothelial-like cells. Although precise factors and mechanisms should be further evaluated, these results suggest that some factors from lens epithelial cells and corneal stroma cells may affect corneal endothelial cell development.

**Table 1:** The summary of Zhang’s methods (2014). *FM* fibroblast differentiation medium, *EM* corneal endothelial cell differentiation medium, *KSR* knockout serum replacement, *FBS* fetal bovine serum, *B27* B27 supplement, *EB* embryoid body

Steps	EB culture	→	Co-culture with corneal stroma cells	
Medium	DMEM/F12, 20% KSR, bFGF (8 ng/ml) etc.		FM: DMEM/F12, B27, EGF (20 ng/ml), bFGF (40 ng/ml), 10% FBS	EM: FM + LECCM (FM:LECCM = 3:1)
Coating	Low adherence culture dish		Fibronectin, laminin, heparin sulfate-coated dish	
Duration	7 days		5 days	2 weeks

#### All-trans retinoic acid and LECCM

Chen et al. derived corneal endothelial-like cells from mouse ES cells and mouse iPS cells by all-trans retinoic acid and LECCM [14]. Table 2 shows the summary of their methods. LECCM was obtained from rabbit lens epithelial cell culture medium. EB culture with 1 μM all-trans retinoic acid promotes neural crest cell differentiation with high expression of NCCs’ markers (Slug, Sox10, p75, etc.). At the second stage differentiation, LECCM derived corneal endothelial-like cells from NCCs. Their corneal endothelial-like cells express Na,K-ATPase, ZO-1, N-cadheirn, Aquaporine-1, etc. Similar to Zhang’s method, LECCM has an important role in the final step of corneal endothelial cell derivation as well, and retinoic acid may have some effect, especially on early stage of corneal endothelial development.

**Table 2:** The summary of Chen’s methods (2015). IMDM; Iscove’s modified Dulbecco’s medium. N2; N2 supplement

Steps	EB culture		→	LECCM culture
Medium	IMDM, 15% FBS, etc.	IMDM, 15% FBS, all-trans retinoic acid (1 μM), etc.		LECCM (DMEM/F12, N2, B27, bFGF (20 ng/ml), ascorbic acid, etc.)
Coating	Low adherence culture dish			Gelatin-coated dish
Duration	4 days	4 days		7 days

#### Dual Smad inhibition and Wnt inhibition

The corneal endothelium derivation method by McCabe et al. was a two-step generation procedure but chemically more defined than previous methods [15]. Since TGF beta, bone morphogenetic protein (BMP), and Wnt are related to EM-T process, regulation of these signals may be important for ME-T process in corneal endothelial development. Table 3 shows the summary of their methods. NCCs were derived from ES cells at the first step with TGF beta signaling blocker (SB431542) and Noggin. Both TGF beta-Smad-2/3 signaling and BMP-Smad-1/5/8 signaling were blocked, and therefore, the procedure was called “dual Smad inhibition” [16]. NCCs with NGFR, SOX10, and FOXC1 expression could be derived from ES cells by chemically defined condition. Next, platelet-derived growth factor B (PDGF-BB), Dickkopf-related protein 2 (DKK-2), and bFGF were able to generate hexagonal corneal endothelial-like cells. DKK-2 is an antagonist of Wnt/beta-catenin signaling. Their corneal endothelial-like cells express Na,K-ATPase, ZO-1, and type VIII collagen (COL8A1), which is the component of Descemet’s membrane. DNA microarray analysis revealed a close similarity between their corneal endothelial cells and primary cultured human corneal endothelial cells. In addition, Wagoner et al. were able to derive corneal endothelial-like cells from iPS cells by modified McCabe’s protocol [17].

**Table 3:** The summary of McCabe’s methods (2015)

Steps	Dual Smad inhibition	→	Cornea media
Medium	DMEM/F12, 20%KSR, SB431542 (10 mM), NOGGIN (500 ng/ml), bFGF (8 ng/ml)		DMEM/F12, 20%KSR, PDGF-BB (10 ng/ml), DKK-2 (10 ng/ml), bFGF (8 ng/ml)
Coating	Matrigel-coated well		Matrigel-coated well
Duration	3 days		14 days

#### Dual Smad inhibition, Wnt inhibition/activation, and ROCK inhibition

Zhao and Afshari also derived corneal endothelial-like cell from iPS cells under chemically defined conditions (Table 4) [18]. The method contains three steps; dual Smad inhibition with SB431542 and LDN193189 (BMP signaling blocker) and Wnt inhibition by IWP2 promote eye field stem cell development from iPS cells. These eye field stem cells express eye field transcription factors PAX6, LHX2, RAX, SIX3, and SIX6. Next, NCCs with HNK-1 and p75NTR expression could be developed from eye field stem cells by canonical Wnt signaling activator CHIR99021. At the last step, SB431542 and ROCK inhibitor H-1125 were able to derive corneal endothelial-like cells from NCCs. Their corneal endothelial-like cell expressed Na,K-ATPase, ZO-1, and N-cadherin. The characteristics of their procedure is tracing complicated EM-T (Wnt activation) and ME-T (Wnt and Smad inhibition) process in corneal endothelial cell development by several small molecule compounds, rather than recombinant proteins. These small molecule compounds may enable reduction of production costs.

**Table 4:** The summary of Zhao’s methods (2016)

Steps	Eye field stem cells differentiation	→	Ocular neural crest stem cells differentiation	→	Corneal endothelial cell induction
Medium	DMEM/F12, N2, B27, SB431542 (5 μM), LDN193189 (50 nM), IWP2 (1 μM), bFGF (20 ng/ml), etc.		DMEM/F12, N2, B27, CHIR99021 (3 μM), 2-phospho-L-ascorbic acid (0.3 mM), etc.		HE-SFM, 5% FBS, SB431542 (1 μM), H-1125 (2.5 μM), 2-phospho-L-ascorbic acid (0.3 mM), etc.
Coating	Matrigel-coated well		Matrigel-coated well		FNC coating mix
Duration	2 days		~ 80% confluence		1 week

#### Self-formed ectodermal autonomous multi-zone method

Hayashi et al. demonstrated the generation from human induced pluripotent stem cells of a self-formed ectodermal autonomous multi-zone (SEAM) of ocular cells [19]. SEAM mimics whole-eye development because cell location within different zones is indicative of lineage, spanning the ocular surface ectoderm, lens, neuro-retina, and retinal pigment epithelium [19]. Interestingly, although SOX10+/p75+ neural crest cells were also found to have emerged in satellite spheres [19], the population of corneal endothelial-like cells seemed to be absent in SEAM. Additional trigger may be required for corneal endothelial cell development from neural crest cells in SEAM.

Table 5 summarizes more detail of the reviewed methods, including cell source and strain (ES or iPS cells, mouse or human), quality check, and in vivo transplant methods.

**Table 5:** Summary of details of reviewed methods, including cell source and strain (ES or iPS cells, mouse or human), markers for cell sorting, quality check experiments, and in vivo transplant methods

	Zhang et al. (2014) [13]	Chen et al. (2015) [14]	McCabe et al. (2015) [15]	Zhao and Afshari (2016) [18]
Cell source	Human ES cells	Mouse ES cells and iPS cells	Human ES cells	Human ES cells and iPS cells
Sorting	Vimentin/N-cadherin double positive cells (7.68%)	N. A.	N. A.	N. A.
Quality check	Real-time PCR (ATP1A1, ATP1B1)	Immunostaining and real-time PCR (Na, K-ATPase, N-cadherin, ZO-1, Aquaporine-1, Vimentin, VE-cadherin, etc.)	Real-time PCR (COL8A1, AQP1), immunostaining (ZO-1, ATP1A1)	Real-time PCR (ATP1A1), immunostaining (ATP1A1, ZO1, N-cadherin)
In vivo transplant model	Rabbit, sheet transplantation	N. A.	N. A.	N. A.

*N. A*. not analyzed

### Challenges for the future

Year by year, the methods have been improved and chemically more defined, which were helpful in not only improving repeatability, but also revealing background mechanisms in corneal endothelium development (Fig. 1). Especially, TGF beta, BMP, or WNT signaling regulation commonly played important roles in the reviewed methods, so EM-T and ME-T process may be the key steps for corneal endothelial cell development. LECCM may include molecules with these effects; however, chemically defined recombinant proteins or small molecule compounds would be more desirable for the purpose of clinical application. Small molecule compounds may also have a merit to reduce product cost.

These improvements may have corneal endothelial regenerative medicine from iPS/ES cells more realistic in the near future. Especially, corneal endothelial regenerative medicine is thought to be very compatible with allogenic iPS/ES cell source, because of anterior chamber-associated immune deviation. Compared to autologous regenerative medicine, allogenic iPS/ES cell source will be able to reduce the cost for cell products.

However, in order to realize regenerative medicine of bullous keratopathy, there are still many problems to be solved. First, there are still no established specific corneal endothelial cell markers, so the markers used vary in each report. Na,K-ATPase expression as pump function marker and ZO-1 expression as tight junction marker are at least necessary as corneal endothelial cells, and many reports have covered them. However, these two markers are not specific for corneal endothelial cells. Corneal endothelial specific markers which, if possible, link corneal endothelial characteristics or functions would be desired.

**Fig. 2:**
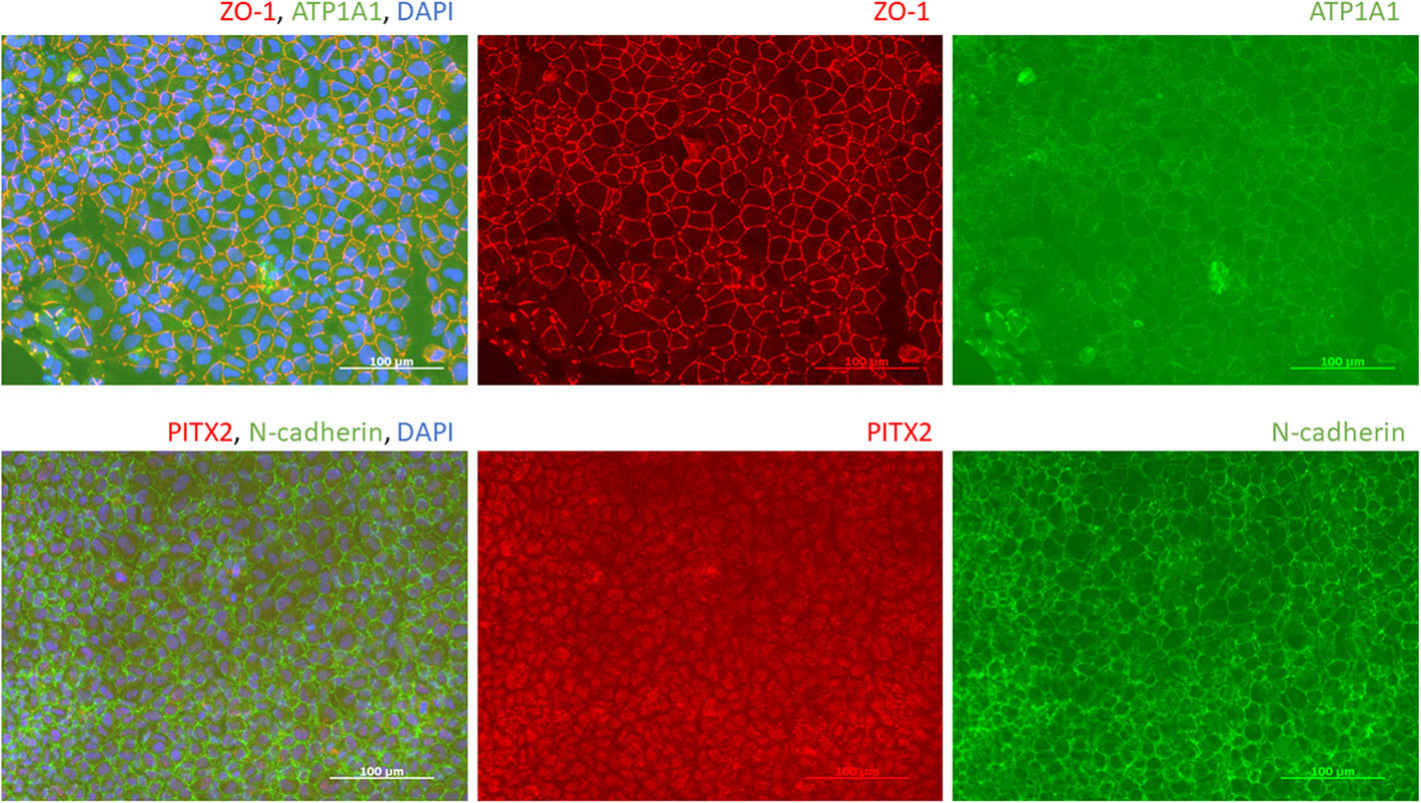
Corneal endothelial-like cell from our lab. Na,K-ATPase alpha-1 subunit (ATP1A1), ZO-1, and N-cadherin express on the cell border, and PITX2 express on cell nuclei

Next, some animal origin materials, such as fetal bovine serum and Matrigel, have still been used in these methods. Component information of other materials, such as human endothelial serum-free medium (HE-SFM) and knockout serum replacement (KSR), are not fully disclosed. The effect of these materials may mask true mechanisms. In addition, the inter-lot difference of animal origin materials may have influenced on products’ repeatability. Recently, we have succeeded in corneal endothelial-like cell production by our original methods with totally animal-free, chemically defined materials (unpublished data), which may be more suitable for clinical trial compared to previous methods. These cells appear cobblestone morphology and express Na, K-ATPase alpha-1 subunits, ZO-1, N-cadherin on cell borders, and PITX2 in cell nuclei (Fig. 2). Comparing four reviewed methods, our corneal endothelial-like cells are unique which completed these four corneal endothelial cell markers.

Lastly, adequate animal models to prove corneal endothelial-like cell function and safety are desired. Zhang et al. performed corneal endothelial-like cell sheet transplantation in rabbit eyes. However, clinically applicable scaffold will be necessary for cell sheet transplantation. The difficulty is that such a scaffold itself is required to keep transparency after transplantation. On the other hand, cell injection methods into rabbit eyes have other difficulties since the anterior chamber space of rabbits is very narrow. Kinoshita et al. proved cultured corneal endothelial cell function by cell injection into the monkey bullous keratopathy model eyes [4]. Primate animal model has a merit that the anterior chamber space is wider than rodents; however, such primate animal model requires much higher costs. In addition, not only the proof of cell function, but also the proof of safety by the animal study would be necessary, especially for the products made from ES or iPS cells.

## Conclusion

Representative methods for corneal endothelial-like cell derivation from ES or iPS cells have been reviewed. The components in the methods have been shifted from animal origin materials to recombinant cytokines and small molecule compounds year by year. Although there still are unknown mechanisms involved, such improvements may enable to reveal developmental process of corneal endothelial cell more clearly in the near future. Efficacy and safety test with adequate animal models will be the challenge for the future.

## Data Availability

Please contact the authors for data requests.

## Abbreviations

bFGF: Basic fibroblast growth factor
DKK-2: Dickkopf-related protein 2
DMEK: Descemet’s membrane endothelial keratoplasty
DSAEK: Descemet’s membrane stripping automated endothelial keratoplasty
EB: Embryoid bodies
EGF: Epidermal growth factor
EM: Corneal endothelial cell differentiation medium
EM-T: Epithelial-mesenchymal transition
ES cells: Embryonal stem cells
FBS: Fetal bovine serum
FM: Fibroblast differentiation medium
HCEC: Human corneal endothelial cells
HE-SFM: Human endothelial serum-free medium
IMDM: Iscove’s modified Dulbecco’s medium
iPS cells: Induced pluripotent stem cells
KSR: Knockout serum replacement
LECCM: Lens epithelial cell-conditioned medium
ME-T: Mesenchymal-endothelial transition
NCCs: Neural crest cells
PDGF-BB: Platelet-derived growth factor B
PKP: Penetrating keratoplasty
POM: Periocular mesenchyme
ROCK: Rho-associated kinase
TGF beta: Transforming growth factor beta
